# Comparison of paracetamol and diclofenac prescribing preferences for adults in primary care

**DOI:** 10.1017/S1463423621000797

**Published:** 2021-12-02

**Authors:** Dilara Bayram, Volkan Aydin, Abdullah Sanli, Mustafa Naci Abanoz, Busra Sibic, Sedat Pala, Omer Atac, Ahmet Akici

**Affiliations:** 1 Department of Pharmacology, Faculty of Pharmacy, Acibadem Mehmet Ali Aydinlar University, İstanbul, Turkey; 2 Department of Medical Pharmacology, International School of Medicine, Istanbul Medipol University, Istanbul, Turkey; 3 School of Medicine, Marmara University, Istanbul, Turkey; 4 Department of Public Health, School of Medicine, Medipol University, Istanbul, Turkey; 5 Department of Medical Pharmacology, School of Medicine, Marmara University, Istanbul, Turkey

**Keywords:** analgesic, diclofenac, pain, paracetamol, prescribing, primary care

## Abstract

**Introduction:**

The most frequently prescribed analgesic drugs in primary care centers in Turkey are diclofenac and paracetamol, respectively. In this study, we aimed to compare paracetamol-included prescriptions (PIP) and diclofenac-included prescriptions (DIP) generated for adult patients in primary care.

**Methods:**

In this cross-sectional study, PIPs (*n* = 280 488) and DIPs (*n* = 337 935) created for adults by systematic sampling among primary care physicians working in Istanbul in 2016 (*n* = 1431) were examined. The demographic characteristics, diagnoses, and additional drugs in PIPs and DIPs were compared.

**Results:**

Women constituted the majority in both groups (69.8% and 67.9%, respectively; *P* < 0.05), and mean age at PIP (52.6 ± 18.8 years) was lower compared to DIP (56.3 ± 16.1 years), (*P* < 0.05). In single-diagnosis prescriptions, 11 of the 15 most common diagnoses in PIP were respiratory tract infections (47.9%); three pain-related diagnoses formed 4.6% of all these prescriptions. In DIP, the number of pain-related diagnoses, mostly of musculoskeletal origin, was eight (28.5%); four diagnoses (7.8%) were upper respiratory tract infections. While hypertension was the third most common diagnosis in PIP (6.1%), it was ranked first in DIP (8.0%). The percentage of prescriptions with additional analgesic (14.0% versus 18.3%, *P* < 0.001), proton-pump inhibitor (13.8% versus 18.4%; *P* < 0.001), and antihypertensive (22.0% versus 24.8%, *P* < 0.001) was lower in PIP compared to DIP. However, the percentage of prescriptions with antibiotics (31.3% versus 14.7%, *P* < 0.001) was higher in PIP.

**Conclusion:**

Paracetamol appears to be preferred mostly in upper respiratory tract infections compared to the preference of diclofenac rather in painful/inflammatory musculoskeletal conditions. The presence of hypertension among the most commonly encountered diagnoses for these analgesic drugs points to challenges in establishing the diagnosing-treatment match and indicates potential irrational prescribing practice, especially for interactions.

## Introduction

Investigation of commonly prescribed drugs in prescriptions issued in primary care can provide important clues about health indicators of the population, as well as providing information about drug use in certain indications. Family health centers, which form approximately one-third of the provision of health care in Turkey, serve to all segments of the society at the primary level with broad range indications (Peksu & Şahin, 2020). A recent nationwide study reported that diclofenac ranked third and paracetamol ranked fourth among the most commonly prescribed drugs in primary care (Bayram *et al.*, 2020). Diclofenac is a powerful non-steroidal anti-inflammatory drug (NSAID) with analgesic and antipyretic action. NSAIDs are widely used in the treatment of diseases with pain, fever, and inflammation (Amadio *et al.*, 1993; Suleyman *et al.*, 2007). Diclofenac is effective in painful and inflammatory conditions, especially in rheumatic diseases (Davies *et al.*, 2000; Khanna *et al.*, 2012). The availability of many pharmaceutical form – oral, intramuscular, intravenous, transdermal, and rectal – and no requirement for dose adjustment in the elderly or patients with renal impairment are among the leading reasons for the widespread use of diclofenac (Todd & Sorkin, 1988). This drug is also frequently used in primary health care centers. For example, in a study conducted in Germany in 2014, it was reported that 61% of physicians prescribing diclofenac were general practitioners. Paracetamol is another analgesic/antipyretic drug that is recommended in the first place in many indications and widely is used. In fact, it is included in thousands of preparations in the world today, and more than 300 preparations contain paracetamol in Turkey (Emet & Yayla, 2016). Having no pronounced anti-inflammatory effect but relatively low incidence of side effects, paracetamol can be particularly preferred in cases where NSAIDs cannot be used – high-risk patient groups, bronchial asthma, peptic ulcer disease, etc. (Hyllested *et al.*, 2002; Jozwiak-Bebenista & Nowak, 2014). It also has rectal and intravenous forms, besides those suitable for oral use like tablets or syrup.

Being one of the most populous metropolis in the world, Istanbul hosts 18.5% of the population of Turkey (14.8 million people) and 17.4% of the primary care physicians in the country work in this city (Başara *et al.*, 2019; Turk Stat, 2016). These physicians generate these prescriptions in the electronic medium, which can be monitored by the health authority with the Prescription Information System (PIS) software (Koyuncuoglu *et al.*, 2017). It would be reasonable to examine the demographic and clinical details of the prescriptions with analgesics, which are among the most frequently used drugs. Whether these drugs, which may be considered as important variables in the health indicators of the society, are used rationally or not should be examined in terms of pharmacoepidemiologic aspects. In this study, we aimed to compare the details of paracetamol-included prescriptions (PIP) and diclofenac-included prescriptions (DIP) written for adult patients in primary care centers throughout Istanbul.

## Methods

In this cross-sectional study, electronic prescriptions issued in primary care centers in Istanbul between 01.01.2016-31.12.2016 and registered in PIS were analyzed after being anonymized. Approval for the study was obtained from the Istanbul Medipol University Non-Interventional Research Ethics Committee (Decision No: 218).

Among the 4,293 primary care physicians registered in Istanbul as of the year 2016, the sample size was calculated as a minimum of 353 by accepting 95% confidence level, 5% margin of error, and 50% incidence. We selected 1431 physicians by systematic sampling with all their prescriptions for adults (≥18 year old) during the year (*n* = 4 678 164). Subsequently, 280 488 PIP and 337 935 DIP were included in the analysis of the study. Patients in these groups were examined based on their sex, age groups (‘18–44 years’, ‘45–64 years’, and ‘≥65 years’), indications, and comorbid diseases (per ICD-10 codes). Drugs were classified according to their ATC codes. The number of drugs per prescription, other accompanying drugs, temporal distribution of prescriptions, and cost were also examined in drug-related analyses.

### Statistical analysis

Statistical analysis was done with GraphPad Prism 5.0 software. Data were expressed as numbers and percentages for categorical variables or mean and standard deviation for continuous variables. The normality of continuous variables was assessed through D’Agostino & Pearson omnibus test, which showed all examined parameters to be distributed normally. Chi-square test and *t*-test were used to compare categorical and continuous variables of the groups, respectively. We used an overall 5% of type I error level to infer statistical significance.

## Results

Women constituted the majority in both groups, and the percentage of prescriptions prescribed for women was higher in PIP compared to DIP (69.8% and 67.9%, respectively; *P* < 0.05). The mean age was lower in PIP (52.6 ± 18.8) than in DIP (56.3 ± 16.1), (*P* < 0.05). More pronounced in women, the number of drugs per prescription is higher in DIP than in PIP (3.90 ± 2.32 and 3.80 ± 2.25, respectively; *P* < 0.05). The highest percentage of patients in PIP and DIP was in the ‘18–44 years old’ (35.5%) and ‘45–64 years old’ (44.3%) groups, respectively. Only PIPs showed a declining trend with increasing age (Table 1). The cost of treatment per prescription was significantly lower in PIP (US$30.6 ± 88.2) compared to that in DIP (US$33.6 ± 70.3; *P* < 0.05). Paracetamol cost per prescription was US$0.8 ± 1.3 in PIP, while diclofenac cost per prescription was US$2.5 ± 5.0 in DIP.

**Table 1:** Comparison of the percentages of prescriptions and the number of drugs per prescriptions in PIPs and DIPs by gender and age groups.

		PIP	DIP	*P*-value
Gender				
Female	Prescription, *n* (%)	195 653 (69.8)	229 520 (67.9)	<0.05
	Drugs per encounter	3.79 ± 2.17	3.94 ± 2.25	<0.05
Male	Prescription, *n* (%)	84 827 (30.2)	108 406 (32.1)	<0.05
	Drugs per encounter	3.84 ± 2.44	3.80 ± 2.45	<0.05
Total^*^	Prescription, *n* (%)	280 480 (100.0)	337 926 (100.0)	–
	Drugs per encounter	3.80 ± 2.25	3.90 ± 2.32	<0.05
Age group				
18–44 years	Prescription, *n* (%)	99 570 (35.5)	78 950 (23.4)	<0.05
	Drugs per encounter	3.22 ± 1.99	3.32 ± 2.33	<0.05
45–64 years	Prescription, *n* (%)	98 484 (35.1)	149 792 (44.3)	<0.05
	Drugs per encounter	3.94 ± 2.19	3.90 ± 2.16	<0.05
≥65 years	Prescription, *n* (%)	82 434 (29.4)	109 192 (32.3)	<0.05
	Drugs per encounter	4.34 ± 2.46	4.32 ± 2.42	>0.05
Total	Prescription, *n* (%)	280 488 (100.0)	337 934 (100.0)	–
	Drugs per encounter	3.80 ± 2.25	3.90 ± 2.32	<0.05

PIP, paracetamol-included prescription; DIP, diclofenac-included prescription.

* Eight prescriptions without gender information in both groups were not included in the total number.

Data are given as mean ± SD.

**Figure 1:**
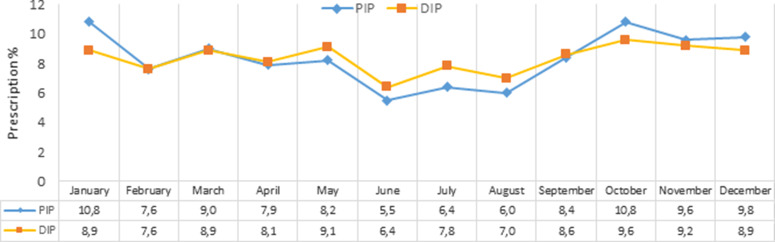
Monthly distribution of PIPs and DIPs. PIP, paracetamol-included prescription; DIP, diclofenac-included prescription.

The season with the highest prescription percentage in both groups was autumn. It was observed that the distribution of PIR and DIR percentages by months was generally parallel to each other throughout the year. Nevertheless, diclofenac was more commonly prescribed in summer and paracetamol in winter (Figure 1).

The total number of diagnoses was 647 795 in PIP and 871 931 in DIP. The number of diagnoses per prescription was 2.31 ± 1.53 and 2.58 ± 1.58 respectively, (*P* < 0.05). PIPs and DIPs featured essential hypertension (9.2% and 9.3%, respectively) and myalgia (3.7% and 5.7%, respectively) as the most frequent indications (Table S1 in Supplementary Appendix). Single diagnoses formed 38.7% of PIPs and 29.8% of DIPs. We determined that 11 of the most commonly encountered 15 diagnoses in the paracetamol group in single-diagnosis prescriptions were respiratory tract infections. We estimated that these 11 diagnoses collectively constituted 47.9% of all single-diagnosis prescriptions. Among the rest, three pain-related diagnoses constituted 4.6%. In DIP, eight pain-related diagnoses in the top 15 constituted 28.5% of all prescriptions and mostly belonged to the musculoskeletal system. Four diagnoses in DIP were accepted as upper RTI and constituted 7.8%. ‘Primary hypertension’ (8.0%), which ranked first in DIP, was the third most common diagnosis (6.1%) in PIP (Table 2).

**Table 2:** The rank and distribution of the diagnoses in PIPs and DIPs containing single diagnoses

Rank	PIP		DIP	
	Diagnose (ICD-10)	*n* (%)	Diagnose (ICD-10)	*n* (%)
1	Acute URTI, u. (J06.9)	12.807 (11.8)	Primary hypertension (I10)	8.088 (8.0)
2	URTI,u. (J39.9)	7.494 (6.9)	Myalgia (M79.1)	7.048 (7.0)
3	Primary hypertension (I10)	6.642 (6.1)	Low back pain (M54.5)	5.212 (5.2)
4	Acute pharyngitis, u. (J02.9)	6.004 (5.5)	Myalgia, u. (M79.19)	3.744 (3.7)
5	Acute nasopharyngitis (J00)	5.858 (5.4)	Lumbago with sciatica (M54.4)	3.238 (3.2)
6	Acute tonsillitis, u. (J03.9)	5.032 (4.6)	Acute URTI, u (J06.9)	2.994 (3.0)
7	Acute bronchitis, u. (J20.9)	3.381 (3.1)	Pain, u. (R52.9)	2.912 (2.9)
8	Acute sinusitis, u. (J01.9)	3.337 (3.1)	Dorsalgia (M54)	2.645 (2.6)
9	Acute LRTI, u. (J22)	2.425 (2.2)	Myalgia, other (M79.18)	2.569 (2.6)
10	Acute pharyngitis (J02)	2.323 (2.1)	Acute nasopharyngitis (J00)	2.078 (2.1)
11	Headache (R51)	1.937 (1.8)	GERD without esophagitis (K21.9)	1.862 (1.9)
12	Other diseases of upper respiratory tract (J39)	1.701 (1.6)	URTI,u (J39.9)	1.428 (1.4)
13	Acute tonsillitis (J03)	1.692 (1.6)	Acute pharyngitis, u. (J02.9)	1.358 (1.3)
14	Pain, u. (R52.9)	1.505 (1.4)	General medical examination (Z00.0)	1.344 (1.3)
15	Myalgia (M79.1)	1.487 (1.4)	Headache (R51)	1.320 (1.3)
Other		45.031 (41.4)		52.785 (52.5)
Total		108.656 (100.0)		100.625 (100.0)

PIP, paracetamol-included prescription; DIP, diclofenac-included prescription; URTI, upper respiratory tract infection; LRTI, lower respiratory tract infection; u, unspecified; GERD, gastro-esophageal reflux disease.

**Figure 2:**
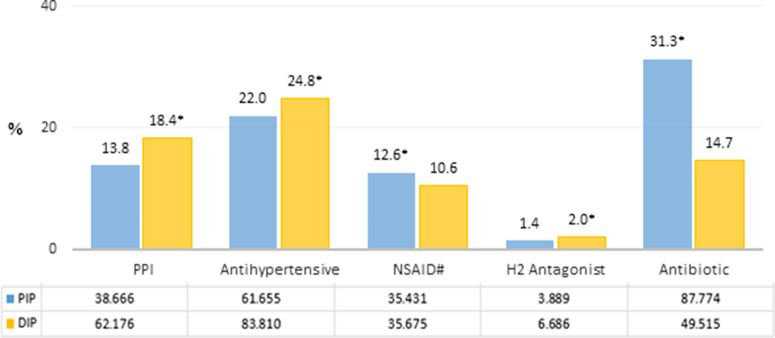
Comparison of the number/percentage distributions of certain prescribed drug groups in PIPs and DIPs. PIP, paracetamol-included prescription; DIP, diclofenac-included prescription; PPI (ATC-4): A02BC; antihypertensive (ATC-2): C02, C03, C07, C08, C09; NSAID (ATC-3): M01A; H2 antagonist (ATC-4): A02BA; antibiotic (ATC-2): J01. #, diclofenac (ATC-5: M01AB05) is excluded in DIP group. *, PIP versus DIP *P* < 0.001.

The percentage of prescriptions containing proton-pump inhibitor (PPI), (13.8% versus 18.4%; *P* < 0.001), H2 receptor antagonist (1.4% versus 2.0%, *P* < 0.001), and antihypertensive (22.0% versus 24.8%, *P* < 0.001) was found to be lower in PIP. On the other hand, the percentage of prescriptions containing NSAIDs (12.6% versus 10.6%, *P* < 0.001) and antibiotics (31.3% versus 14.7%, *P* < 0.001) was higher in PIP than in DIP (*P* < 0.001), (Figure 2). The cost of PPIs per prescription in DIP was US$17.3 ± 9.4. The top most frequently encountered drug group at ATC-3 level was ‘peptic ulcer and gastro-esophageal reflux drugs (A02B)’ in PIP (6.2%) and ‘centrally acting muscle relaxants (M03B)’ in DIP (8.4%), (Table S2 in Supplementary Appendix).

‘Other cold preparations’ (4.0%) was the most commonly prescribed drug in PIP and ranked second in DIP (3.3%). While thiocolchicoside was the most frequently prescribed drug in DIP (3.5%), it was not found to be listed among the first fifteen mostly co-prescribed drugs in PIP. The second most commonly co-prescribed drug was ‘amoxicillin and beta-lactamase inhibitor combinations’ (3.5%) in PIP (Table 3).

**Table 3:** The distribution of the numbers and percentages of the top 15 most frequently encountered drugs in PIPs and DIPs

PIP			DIP	
Rank	Drug (ATC-5)	Drug, *n* (%)	Drug (ATC-5)	Drug, *n* (%)
1	Other cold preparations (R05X)	42.229 (4.0)	Thiocolchicoside (M03BX05)	46.417 (3.5)
2	Amoxicillin and beta-lactamase inhibitor (J01CR02)	37.270 (3.5)	Other cold preparations (R05X)	43.189 (3.3)
3	Various (A01AD11)	26.013 (2.4)	Acetylsalicylic acid (B01AC06)	22.383 (1.7)
4	Acetylsalicylic acid (B01AC06)	17.648 (1.7)	Lansoprazole (A02BC03)	21.787 (1.7)
5	Acetylcysteine (R05CB01)	15.372 (1.4)	Amoxicillin and beta-lactamase inhibitor (J01CR02)	19.502 (1.5)
6	Lansoprazole (A02BC03)	13.582 (1.3)	Metformin (A10BA02)	18.947 (1.4)
7	Diclofenac (M01AB05)	13.012 (1.2)	Pantoprazole (A02BC02)	18.219 (1.4)
8	Metformin (A10BA02)	12.103 (1.1)	Topical diclofenac (M02AA15)	16.609 (1.3)
9	Metoprolol (C07AB02)	11.816 (1.1)	Vitamin B1 comb. (A11DB)	15.913 (1.2)
10	Pantoprazole (A02BC02)	11.809 (1.1)	Imidazoles/triazoles in comb. with corticosteroids (D01AC20)	15.266 (1.2)
11	Benzydamine (A01AD02)	11.302 (1.1)	Esomeprazole (A02BC05)	14.156 (1.1)
12	Vitamin B1 comb. (A11DB)	10.511 (1.0)	Levothyroxine sodium (H03AA01)	14.075 (1.1)
13	Levothyroxine sodium (H03AA01)	10.229 (1.0)	Metoprolol (C07AB02)	13.853 (1.1)
14	Flurbiprofen (R02AX01)	10.203 (1.0)	Paracetamol (N02BE01)	12.873 (1.0)
15	Butamirate (R05DB13)	9.808 (0.9)	Other non-therapeutic auxiliary products (V07AY)	12.616 (1.0)
First 15 Drug Total		252.907 (32.2)	First 15 Drug Total	305.805 (31.4)
Other Drugs		532.768 (67.8)	Other Drugs	668.784 (68.6)
Total		785.675 (100.0)	Total	974.589 (100.0)

PIP, paracetamol-included prescription; DIP, diclofenac-included prescription; comb., combination.

Seasonal distribution of antibiotic co-prescriptions was similar to that observed for overall PIP and DIP, showing slight predominance of the former in winter and the latter in summer months (Figure S1 at Supplementary Appendix).

## Discussion

In this study, paracetamol and diclofenac, which are frequently prescribed drugs in primary care medicine, were evaluated in terms of age, gender, indications, and comorbidities. In addition, these drugs were analyzed for possible interactions with other prescription drugs.

Our study where we examined paracetamol and diclofenac prescriptions in primary care stands out RTIs for paracetamol and painful/inflammatory musculoskeletal conditions for diclofenac use. Besides, high number of accompanying drugs, especially PPIs and antihypertensives, and the high average prescription cost in diclofenac could be regarded as remarkable as well as the age-related decreasing trend in paracetamol use.

In many studies about health care and drug use at both global and national level, health care utilization was reported more common in women (Bayram *et al.*, 2020; Glaeske *et al.*, 2012; Wändell *et al.*, 2013). This seems to be also preserved for paracetamol and diclofenac use in our study, considering the fact that slightly above 2/3 of prescriptions were generated for women in both groups. On the other hand, we observed several differences in terms of age groups. In two separate studies in France where PIPs and NSAIDs were examined, the average age of the patients was reported as 48.3 and 47.0 years, respectively; and in another study conducted in Norway, the average age of those using diclofenac was 44.0 years (Duong *et al.*, 2013; Duong *et al.*, 2016; Hasford *et al.*, 2004). The higher average age of PIP and DIPs in our study (>50 years) can be attributed to the fact that our sample included only adult patients. It is known that the need for anti-inflammatory activity in addition to analgesia is increasing gradually in musculoskeletal conditions such as osteoarthritis, myalgia (Zhang *et al.*, 2004; Elewaut, 2005). Considering that such diseases as osteoarthritis or rheumatoid arthritis are more common at the age of over 45 years, the higher preference of diclofenac in the ‘45–64 age’ group compared to paracetamol in our study may be associated with the prevalence of these clinical conditions (Çakmak *et al.*, 2004; Neogi & Zhang, 2013). Although it may be assumed that the frequency and severity of these diseases and symptoms increase with age, the relative decrease in anti-inflammatory analgesic preference in our study may be associated with other factors limiting the use of NSAIDs. For instance, the vulnerability of elderly population to side effects might have been related to lower preference of diclofenac over paracetamol (Goldstein & Morrison, 2005; Persons, 2009; Arnstein, 2010). In fact, paracetamol was reported as the most common drug in >65-year-old regular analgesic users who applied to a family medicine unit due to musculoskeletal problems (Öksüz *et al.*, 2017). Nevertheless, the fact that diagnoses originating from the musculoskeletal system were not listed among the top in the PIP group may be partly associated with the higher representation of young and middle age groups who applied to primary care in Turkey (Bayram *et al.*, 2020). The literature showed reports of inconsistent results across countries on the use of these drugs in various age groups. Paracetamol prescriptions were reported to decline with increasing age over 45 years of age in France, whereas Scandinavian countries reported highest use in the geriatric age group (Duong *et al.*, 2016; Wastesson *et al.*, 2018). Another study from France also showed decreasing use of NSAIDs over 45 years of age, while its use was reported as the highest in the geriatric age group in the United States and in various countries (Duong *et al.*, 2013; Shaheen *et al.*, 2006; Laine, 2001).

We encountered hypertension as the most common diagnosis in both PIP and DIPs, regardless of having single or multiple indications. This may be partially related to the prevalence of the disease in adult patients as it is one of the most common diseases within the society (NCD Risk Factor Collaboration and McLachlan, 2016; Başara *et al.*, 2021). On the other hand, prescriptions with a single diagnosis can offer more concrete clues in terms of uncovering the drug indication relationship. Accordingly, when the single-diagnosis subgroups were examined, we observed that hypertension remained the most common diagnosis in DIP and ranked third in PIP. Antihypertensive drugs show significant drug interactions with NSAIDs including diclofenac, giving rise to problems such as increased blood pressure and gastrointestinal side effects (Williams *et al.*, 2018). Physicians are expected to be careful about this interaction and avoid it as much as possible when prescribing for hypertensive patients. The higher prevalence of hypertension and concomitant use of antihypertensive drugs in DIP suggests that primary care physicians in our study tend to practice less attention for this interaction. Even if some of the patients require analgesics for various conditions that may be overlooked in prescriptions, this high rate of hypertension indicates that the use of NSAIDs, especially of diclofenac, warrants questioning in primary care.

The predominance of pathologies such as myalgia, dorsalgia, and lumbago in the DIP group seems consistent with the other finding that diclofenac was most commonly co-prescribed with thiocolchicoside. Nonetheless, thiocolchicoside is one of the drugs that have been tried to be restricted to its use in recent years due to safety problems (Kamath, 2013; European Medicines Agency, 2013; Ministry of Health of Turkey, 2014). Such higher co-prescription of a muscle relaxant drug with diclofenac indicates the necessity of making detailed rationality inquiries for each of these indications. Less commonly found in DIP (8%), upper RTIs constituted 11 of the 15 most common diagnoses in the PIP group (48%), supporting paracetamol to be among the most frequently used drugs in the symptomatic treatment of these diseases. In parallel with this finding, paracetamol was reported to be among the most frequently prescribed drugs due to RTIs in a previous study (Akıcı *et al.*, 2014). On the other hand, in our study, it was observed that 31.3% of PIP had antibiotics. A similar study in France reported 35.6% of PIPs to have amoxicillin (Duong *et al.*, 2016). It is known that the paracetamol-based symptomatic treatment of RTI is more frequently performed during the flu/cold season, and antibiotics are often prescribed together with analgesics in the treatment of upper RTIs (Shifmann *et al.*, 2018; Trap & Hansen, 2002). In fact, we observed that paracetamol and diclofenac were mostly prescribed in autumn/winter seasons and the difference between summer and winter months was quite marked for PIP. This provides further support for prescribing paracetamol against upper RTIs. In addition, paracetamol-antibiotic co-prescription was observed to be higher in the autumn/winter months when the infections are common. In Turkey, ‘penicillin and beta-lactamase inhibitor combinations’ are the most frequently prescribed antibiotic group (Isli *et al.*, 2020). In our study, ranking of this group as second in PIPs and tenth in DIPs indicates that this group preserves its prioritized place in primary care also for conditions that require paracetamol or diclofenac, more marked with the former.

It is known that acid-suppressing drugs are often prescribed together with NSAIDs due to their gastroprotective effects (Pettit, 2005; Gwee *et al.*, 2018; Lanza *et al.*, 2009). While the use of PPI should be preferred in very limited clinical situations in order to prevent the NSAID-related gastrointestinal adverse effects, it is argued that this practice is particularly common in Turkey (Bayram *et al.*, 2020; Çelik *et al.*, 2021). Undoubtedly, PPIs can be widely used in some indications beside NSAID-related gastropathies such as peptic ulcer, gastro-esophageal reflux. (Yuan *et al.*, 2016; Sandhu & Fass, 2018). In our study, higher co-prescription of PPI in DIP compared to PIP reflects the tendency of physicians to gastroprotection with PPI when prescribing NSAIDs. Apart from the risk of causing adverse effects and interactions, unnecessary and excessive PPI use may also trigger other irrational drug use problems like increasing medication costs (Freedberg *et al.*, 2017; Farrell *et al.*, 2017). In fact, we determined that the average cost of diclofenac per prescription was 2.5 US$, whereas the average cost of PPI per prescription for these prescriptions was 17.3 US$. Accordingly, addition of a PPI to DIP might be associated with a near 7-fold increment in prescription costs. Moreover, considering overutilization of PPIs beyond gastroprotective purposes implies that a significant part of such cost burden could be regarded as drug wastage. On the other hand, long-term use of PPIs has been associated with various side effects such as the increased risk of infections such as *Clostridium difficile* and pneumonia, dementia, decreased absorption of vitamins and minerals, and chronic kidney disease (Thomson *et al.*, 2010; Eusebi *et al.*, 2017; Jaynes *et al.*, 2018; Schoenfeld & Grady, 2016).

Our study has some limitations. Prescribing behaviors of physicians were examined within the scope of the study, and it should be considered that these prescriptions may not fully reflect the actual usage data of the patients. The diagnoses prescribed by the physicians were accepted as correct, and no additional examinations were made to test the accuracy of these diagnoses. Each prescription was evaluated as belonging to a separate patient and it was accepted that they applied to the primary care for the first time. However, in practice, the fact that some patients may have PIP or DIP more than once during the study period has been neglected. There are various fixed dose combination preparations containing paracetamol or diclofenac active ingredients. Such combinations were not included in the study, and prescriptions containing only paracetamol or diclofenac as active ingredients were examined. On the other hand, the fact that our research universe covers a large metropolis such as Istanbul and that its data are extrapolated in this way offers the opportunity to comment on related analgesic prescriptions throughout the country and elsewhere increases the value of the information that our study will add to the literature.

In conclusion, the prescriptions of most commonly used analgesic drugs in primary care show that paracetamol is mostly preferred in the young-middle age group and diclofenac in the middle-advanced age group. Certain diagnoses such as ‘respiratory tract infections’ in PIP and ‘musculoskeletal diseases’ and ‘hypertension’ in DIP were remarkably common. This reveals that the preference of these two analgesics by physicians may vary depending on the indication and accompanying secondary diseases of the patients. However, the fact that hypertension is at the top of the prescriptions for these drugs with an indication of analgesia, and that it is the most common in the diclofenac group, points to the difficulties in establishing the diagnosis-treatment relationship. Furthermore, it also uncovers irrational prescribing practice by overlooking potential disease-drug interaction between NSAIDs and hypertension. It may be suggested that analgesic-prescribing tendency of primary care physicians towards certain medications or conditions in our study needs to be elaborated with further focused qualitative and/or quantitative studies that involve patient- or physician-centered clinical data.

## Supplementary material

For supplementary material accompanying this paper visit http://dx.doi.org/10.1017/S1463423621000797. click here to view supplementary material
