# Supplementary material for: Comparison of paracetamol and diclofenac prescribing preferences for adults in primary care
Source: Prim Health Care Res Dev. 2021 Dec 2;22:e78. doi: 10.1017/S1463423621000797 (PMC8724224; doi:10.1017/S1463423621000797)
Supplement: Supplementary file 1 [file phcsup.zip › S1463423621000797sup001.docx]

**Table S2.** Distribution of drug groups (ATC-3 level) in PIPs and DIPs.

| Rank | PIP | | | DIP | | |
| --- | --- | --- | --- | --- | --- | --- |
|  | **Drug Group (ATC-3)** | | **n (%*)** | **Drug Group (ATC-3)** | | **n (%*)** |
| 1 | Drugs for peptic ulcer and gastro-oesophageal reflux disease (A02B) | | 48.919 (6.2) | Muscle relaxants, centrally acting agents (M03B) | | 78.329 (8.4) |
| 2 | Beta-lactam antibacterials, penicillins (J01C) | | 48.791 (6.2) | Drugs for peptic ulcer and gastro-oesophageal reflux disease (A02B) | | 77.135 (8.2) |
| 3 | Other cold preparations (R05X) | | 42.229 (5.4) | Topical products for joint and muscular pain (M02A) | | 54.803 (5.9) |
| 4 | Anti-inflammatory and antirheumatic products, non-steroids (M01A) | | 38.352 (4.9) | Other cold preparations (R05X) | | - 1. (4.6) |
| 5 | Stomatological preparations (A01A) | | 38.126 (4.9) | Blood glucose lowering drugs, excl. insulins (A10B) | | 37.688 (4.0) |
| 6 | Expectorants, excl. combinations with cough suppressants (R05C) | | 26.300 (3.4) | Topical antifungals (D01A) | | 28.232 (3.0) |
| 7 | Decongestants and other nasal preparations for topical use (R01A) | | 23.830 (3.0) | Antithrombotic agents (B01A) | | - 1. (3.0) |
| 8 | Antithrombotic agents (B01A) | | 23.530 (3.0) | Antidepressants (N06A) | | 27.509 (2.9) |
| 9 | Blood glucose lowering drugs, excl. insulins (A10B) | | 23.240 (3.0) | Beta blocking agents (C07A) | | 24.751 (2.6) |
| 10 | Topical products for joint and muscular pain (M02A) | | 21.393 (2.7) | Beta-lactam antibacterials, penicillins (J01C) | | 24.666 (2.6) |
| 11 | Cough suppressants, excl. combinations with expectorants (R05D) | | 20.460 (2.6) | Non-opioid analgesic (N02B) | | 23.997 (2.6) |
| 12 | Beta blocking agents (C07A) | | 20.291 (2.6) | Angiotensin II receptor blockers (ARBs), comb. (C09D) | | 22.905 (2.4) |
| 13 | Other beta-lactam antibacterials (J01D) | | 20.186 (2.6) | Antihistamines (R06A) | | 19.504 (2.1) |
| 14 | Topical antifungals (D01A) | | 18.032 (2.3) | ACE inhibitors, comb. (C09B) | | 16.723 (1.8) |
| 15 | Antidepressants (N06A) | | 17.749 (2.3) | Stomatological preparations (A01A) | | 16.404 (1.8) |
| Other | | 351.781 (44.9) | | **Other** | 412.802 (44.1) | |
| Total | | 783.209 (100.0) | | **Total** | 936.333 (100.0) | |

PIP, paracetamol-included prescription; DIP, diclofenac-included prescription; comb., combination.
