# Supplementary material for: Comparison of paracetamol and diclofenac prescribing preferences for adults in primary care
Source: Prim Health Care Res Dev. 2021 Dec 2;22:e78. doi: 10.1017/S1463423621000797 (PMC8724224; doi:10.1017/S1463423621000797)
Supplement: Supplementary file 1 [file phcsup.zip › S1463423621000797sup002.docx]

**Table S1.** The rank and distribution of the diagnoses in all of single and multiple diagnoses-containing PIPs and DIPs.

| Rank | PIP | | | DIP | |
| --- | --- | --- | --- | --- | --- |
|  | **Diagnose (ICD-10)** | | **n (%)** | **Diagnose (ICD-10)** | **n (%)** |
| 1 | Primary hypertension (I10) | | 59.302 (9.2) | Primary hypertension (I10) | 81.120 (9.3) |
| 2 | Myalgia (M79.1) | | 24.067 (3.7) | Myalgia (M79.1) | 49.719 (5.7) |
| 3 | Acute URI, u. (J06.9) | | 23.147 (3.6) | GERD without esophagitis (K21.9) | 23.247 (2.7) |
| 4 | URI,u. (J39.9) | | 16.942 (2.6) | Myalgia, u. (M79.19) | 23.134 (2.7) |
| 5 | Pain, u. (R52.9) | | 16.202 (2.5) | Pain, u. (R52.9) | 22.595 (2.6) |
| 6 | Headache (R51) | | 15.922 (2.5) | GERD (K21) | 19.543 (2.2) |
| 7 | GERD without esophagitis (K21.9) | | 15.509 (2.4) | Myalgia, other (M79.18) | 16.631 (1.9) |
| 8 | GERD (K21) | | 12.693 (2.0) | Low back pain (M54.5) | 16.200 (1.9) |
| 9 | Acute nasopharyngitis (J00) | | 12.569 (1.9) | Dorsalgia (M54) | 12.108 (1.4) |
| 10 | Acute pharyngitis, u. (J02.9) | | 10.462 (1.6) | Acute URI, u. (J06.9) | 11.681 (1.3) |
| 11 | Myalgia, u. (M79.19) | | 10.302 (1.6) | General medical examination (Z00.0) | 11.519 (1.3) |
| 12 | General medical examination (Z00.0) | | 9.690 (1.5) | Pain in joint (M25.5) | 11.030 (1.3) |
| 13 | Myalgia, other (M79.18) | | 8.676 (1.3) | Lumbago with sciatica (M54.4) | 10.444 (1.2) |
| 14 | Acute bronchitis, u. (J20.9) | | 7.479 (1.2) | Dermatitis, u. (L30.9) | 9.714 (1.1) |
| 15 | Acute tonsillitis, u. (J03.9) | | 7.283 (1.1) | Allergy, u. (T78.4) | 9.661 (1.1) |
| Other Diognosis | | | 397.550 (61.4) | **Other Diognosis** | 543.585 (62.3) |
| Total | | 647.795 (100.0) | | **Total** | 871.931 (100.0) |

PIP, paracetamol-included prescription; DIP, diclofenac-included prescription: u., unspecified
