# Supplementary figures and images for: Comparison of paracetamol and diclofenac prescribing preferences for adults in primary care
Source: Prim Health Care Res Dev. 2021 Dec 2;22:e78. doi: 10.1017/S1463423621000797 (PMC8724224; doi:10.1017/S1463423621000797)

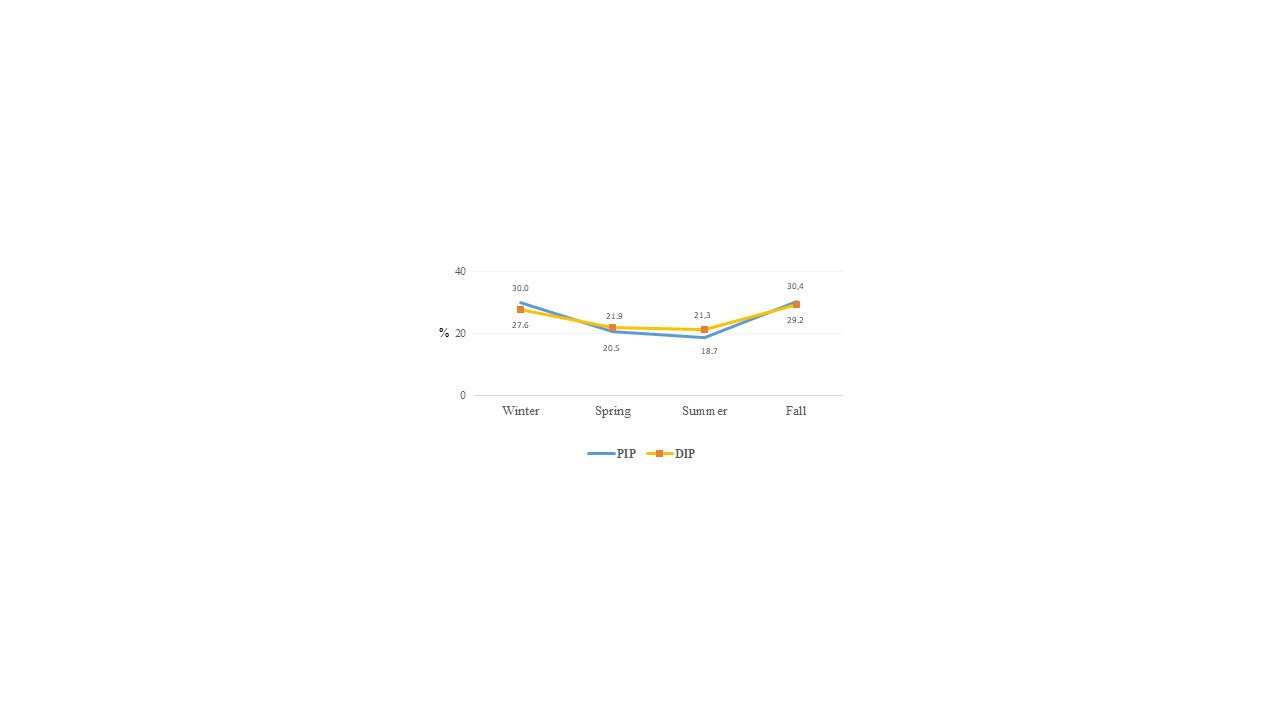

Supplement: Supplementary file 1 [file phcsup.zip › S1463423621000797sup003.tif]
